# Theoretical Study on the Photoemission Performance of a Transmission Mode In_0.15_Ga_0.85_As Photocathode in the Near-Infrared Region

**DOI:** 10.3390/molecules28135262

**Published:** 2023-07-07

**Authors:** Huan Wang, Jiajun Linghu, Pengfei Zou, Xuezhi Wang, Hao Shen, Bingru Hai

**Affiliations:** 1 School of Science, Chang’an University, Xi’an 710061, China; linghujiajun@chd.edu.cn (J.L.); zoupf@chd.edu.cn (P.Z.); xzh_wang@chd.edu.cn (X.W.); haoshen@chd.edu.cn (H.S.); 2 School of Physics, Northwest University, Xi’an 710069, China

**Keywords:** photoemission, first principles calculations, time response characteristics, quantum efficiency

## Abstract

Benefiting from a high quantum efficiency, low thermal emittance, and large absorption coefficient, In_x_Ga_1−x_As is an excellent group III–V compound for negative electron affinity (NEA) photocathodes. As the emission layer, In_x_Ga_1−x_As, where x = 0.15, has the optimal performance for detection in the near-infrared (NIR) region. Herein, an NEA In_0.15_Ga_0.85_As photocathode with Al_0.63_Ga_0.37_As as the buffer layer is designed in the form of a transmission mode module. The electronic band structures and optical properties of In_0.15_Ga_0.85_As and Al_0.63_Ga_0.37_As are calculated based on density functional theory. The time response characteristics of the In_0.15_Ga_0.85_As photocathode have been fully investigated by changing the photoelectron diffusion coefficient, the interface recombination velocity, and the thickness of the emission layer. Our results demonstrate that the response time of the In_0.15_Ga_0.85_As photocathode can be reduced to 6.1 ps with an incident wavelength of 1064 nm. The quantum efficiency of the In_0.15_Ga_0.85_As photocathode is simulated by taking into account multilayer optical thin film theory. The results indicate that a high quantum efficiency can be obtained by parameter optimization of the emission layer. This paper provides significant theoretical support for the applications of semiconductor photocathodes in the near-infrared region, especially for the study of ultrafast responses in the photoemission process.

## 1. Introduction

Photocathodes can emit photoelectrons due to the photoelectric effect, which has been extensively applied in electronic sources [1], photodetectors [2], photocatalytic fuel cells [3], and sensors [4]. Negative electron affinity (NEA) photocathodes exhibit super electron emission under light illumination owing to the fact that the photoelectrons can diffuse to the surface and reach vacuum level (*E_v_*) without extra energy, which leads to a high sensitivity [5,6]. In order to satisfy the high performance of the photoemission process, NEA photocathodes with fast response times, low energy spreads, and high quantum efficiencies (QE) are desirable [7,8]. The past decades have witnessed a huge development of alkali-based materials as NEA photocathodes, such as Ag-O-Cs [9] and alkali halide [10], as well as the alkali antimonide photocathode [11], which have been widely used as electron sources in accelerators and even exhibit spin polarized properties [12]. To further achieve the high requirements of the photoelectric response, searching for new materials is urgently required to achieve excellent photoemission properties.

Recently, group III–V materials have been considered as potential candidates for photocathodes, due to their high quantum efficiency [13], low thermal emittance [14], and fast response time [15]. semiconductor photocathodes with an NEA nature are sensitive to the wavelength of the incident light, which is determined by the band gap energy (*E_g_*). For example, GaAs (*E_g_*~1.43 eV) [16] matching the visible spectrum and GaN (*E*_g_~3.4 eV) [17] serving for ultraviolet light are both typically binary III–V semiconductors for NEA photocathodes. The different band gaps refer to the varying band bending on the semiconductor surface, which accounts for the different states of the electron affinity and photoemission performance. In order to expand the response spectrum of NEA photocathodes, further exploration of semiconductor photocathodes with appropriate properties, including a proper band gap and a high QE, is still mandatory.

Compared with binary III–V semiconductors, In_x_Ga_1−x_As photocathodes have great potential to extend the near-infrared (NIR) response, and their band gap can be flexibly regulated from 0.35 eV to 1.43 eV by changing the ratio of In [18,19]. Correspondingly, the spectral response of In_x_Ga_1−x_As within the range of 0.87~3.54 μm is adjustable, which can perfectly match the spectral range of moonlight (0.4~2.5 μm) as well. Therefore, In_x_Ga_1−x_As photocathodes offer a useful method for realizing applications in image intensifiers [20], ultrafast photodetectors [21], and biomedical monitoring [22] under conditions of weak NIR light. An InGaAs photocathode was first adopted by the US Litton System in the night vision system with a much higher QE in the NIR region than that of the GaAs photocathode [23]. With the development of material growth techniques and the goal of reducing the lattice mismatch of photocathodes, a series of InGaAs-based heterojunction structures with different buffer layers was reported, such as InP/InGaAs [24] and InGaAs/AlGaAs [25]. In addition, doping is a helpful step for the formation of the NEA photocathode, and can also be used to guide the In_x_Ga_1−x_As material growth for the photocathode [26]. Moreover, the NEA activation procedure, in which cesium and oxygen adsorption are alternately supplied on the photocathode surface, can make the surface vacuum level of the NEA photocathodes lower than the conduction band minimum (CBM) of the bulk and result in a high QE [27,28]. Aside from the QE, the response time also plays a significant role in the photoemission process, and has attracted great interest in recent years. Spicer et al. [29] have reported that the photoelectron emission process in NEA photocathodes includes photon absorption, photoelectron transport, and escape from the material surface to the vacuum. During the absorption process, photoemission with photons of energies near the band gap energy can produce a long temporal tail that extends into the range of ~100 ps owing to the thermal emittance and low absorption of near band gap energy photons in the semiconductor photocathode [14]. Egorenkov et al. have proposed that a long response time can render temporal shaping of the laser pulse, leading to ineffective illumination on the surface of the photocathode and a reduction in the photoemission performance [24]. These works demonstrate that the response times of NEA photocathode materials are strongly dependent on the photon energy of the incident light, which is also affected by the electron diffusion length [30] and back interface recombination velocity [31]. In_x_Ga_1−x_As with an In component of 0.15 has been employed as a photoemission layer in a NEA photocathode, which has been verified as the optimal cathode composition for detection at 1064 nm [32]. To the best of our knowledge, the dynamic characteristics of the In_0.15_Ga_0.85_As photocathode have still not been revealed. Consequently, it is highly desired to design novel semiconductor photocathodes with fast response times and systematically evaluate their photoemission properties.

In this paper, a transmission mode In_0.15_Ga_0.85_As photocathode with an Al_0.63_Ga_0.37_As buffer layer is proposed to improve the photoemission properties in the NIR region. The electronic band structures, density of states, and optical properties of In_0.15_Ga_0.85_As and Al_0.63_Ga_0.37_As are analyzed by first principles calculations. By solving the photoelectron diffusion model, the time response dependency of the In_0.15_Ga_0.85_As photocathode on the electron diffusion coefficient, the back interface recombination velocity, and the thickness of the emission layer is fully investigated. The theoretical QE of the In_0.15_Ga_0.85_As photocathode can be deduced by considering the multilayer optical thin film equation, which shows a high photoemission performance in the NIR region. Our works provide a theoretical foundation for designing NEA photocathode structures from the perspective of microscopic atoms, and contribute to the performance improvement of the photoemission process.

## 2. Results and Discussion

### 2.1. Geometrical Structure of In_0.15_Ga_0.85_As and Al_0.63_Ga_0.37_As

**Figure 1:**
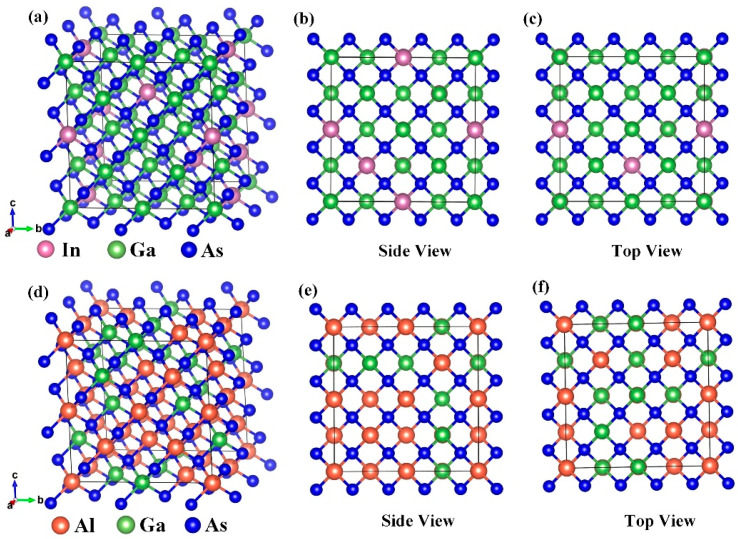
(**a**) Model of In_0.15_Ga_0.85_As crystal cell. (**b**) Crystal structures of In_0.15_Ga_0.85_As in side view and (**c**) top view. (**d**) Model of Al_0.63_Ga_0.37_As crystal cell. (**e**) Crystal structures of Al_0.63_Ga_0.37_As in side view and (**f**) top view.

Derived from the perfect face-centered cubic structure of GaAs, a supercell composed of 2 × 2 × 2 unit cells was created and five Ga atoms were substituted by five In atoms considering the model symmetry. The model of bulk In_0.15_Ga_0.85_As before the geometry optimization is depicted in Figure 1a. Figure 1b,c shows the structures of the bulk In_0.15_Ga_0.85_As from a side view and a top view. Similarly, 20 Ga atoms were replaced by Al atoms to form the supercell of the bulk Al_0.63_Ga_0.37_As. The model of bulk Al_0.63_Ga_0.37_As before the geometry optimization is depicted in Figure 1d. Figure 1e,f shows the structures of the bulk Al_0.63_Ga_0.37_As in side view and top view as well. After geometry optimization, the lattice constants of the bulk In_0.15_Ga_0.85_As and Al_0.63_Ga_0.37_As are 0.567 nm and 0.565 nm, which agrees well with the literature value [33].

### 2.2. Device Design of the Transmission Mode In_0.15_Ga_0.85_As Photocathode

**Figure 2:**
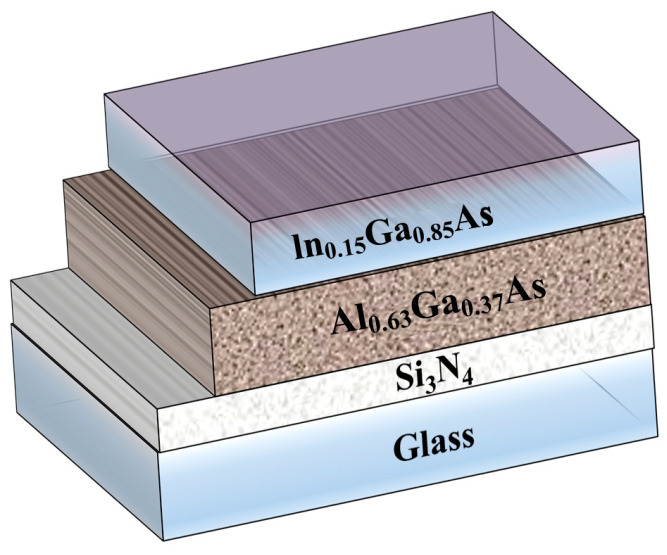
Schematic diagram of the NEA In_0.15_Ga_0.63_As photocathode.

The construction of the transmission mode In_0.15_Ga_0.85_As photocathode with negative electron affinity is shown in Figure 2. A Si_3_N_4_ layer is employed as the anti-reflection film to reduce the energy loss of incident photons. The In_0.15_Ga_0.85_As layer serves as the emission layer to generate photoelectrons in the near-infrared region. Al_0.63_Ga_0.37_As is applied as the buffer layer to prevent photoelectrons excited near the interface from recombining, which can also reduce the mismatch of thermal expansion caused by the thermal bonding between the emission layer material and the glass substrate [34]. 

### 2.3. Band Structures and Density of States (DOS) of In_0.15_Ga_0.85_As and Al_0.63_Ga_0.37_As

**Figure 3:**
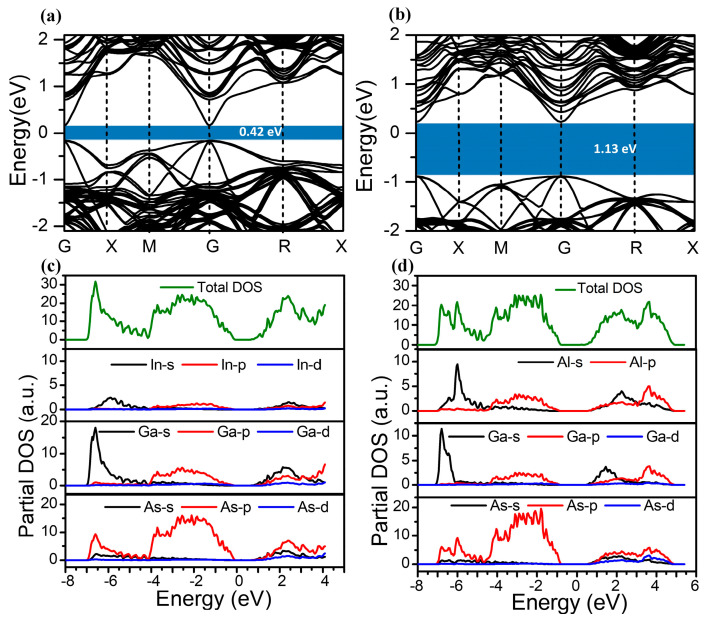
Band structures of (**a**) In_0.15_Ga_0.85_As and (**b**) Al_0.63_Ga_0.37_As. (**c**) DOS results for In, Ga, and As elements in In_0.15_Ga_0.85_As. (**d**) DOS results for Al, Ga, and As elements in Al_0.63_Ga_0.37_As.

The energy bands of In_0.15_Ga_0.85_As are 0.42 eV and 1.13 eV, as shown in Figure 3a,b. These values are smaller than the theoretical values [19] because the DFT adopted for approximate calculation in this work is based on the theory that ground states substitute the excited states, so the energy gaps in calculations are weaker than those in experiments, but this does not influence the analysis of the energy gap. It is indicated that In_0.15_Ga_0.85_As is a direct band gap material, which is beneficial for the excitation and escape of optoelectronics. Nevertheless, Al_0.63_Ga_0.37_As is an indirect band gap material, which is in good agreement with a previous report [35]. Figure 3c presents the density of states (DOS) of In_0.15_Ga_0.85_As, which verifies the position of the valence bands, consisting of a lower valence band with the energy from −7.13 eV to −4.1 eV. The sharp peaks appearing at −6.5 eV are composed of Ga_4s_ and As_4p_ electronic states. The upper valence band (i.e., valence band maximum, VBM) ranging from −4.2 eV to −0.1 eV is induced by the In_5p_, As_4p_, and Ga_4p_ states. However, the conducting band minimum (CBM) located in the energy range between 0.8 eV and 4.1 eV is formed by the Ga_4s_ state and As_4p_ state. The DOS of Al_0.63_Ga_0.37_As is shown in Figure 3d, which demonstrates that the VBM is mainly attributed to Al_3p_, As_4p_, and Ga_4p_ states. The CBM is a mixture of Al_3s_, Al_3p_, Ga_4s_, and Ga_4p_ states, in accordance with previous reports [36,37].

### 2.4. Optical Properties of In_0.15_Ga_0.85_As and In_0.63_Ga_0.37_As

**Figure 4:**
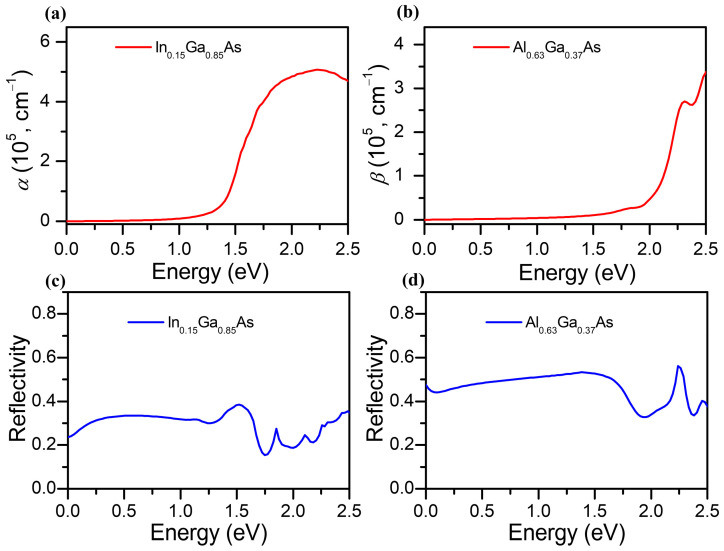
Absorption coefficient of (**a**) In_0.15_Ga_0.85_As and (**b**) Al_0.63_Ga_0.37_As. Reflectivity of (**c**) In_0.15_Ga_0.85_As and (**d**) Al_0.63_Ga_0.37_As.

The absorption coefficient of In_0.15_Ga_0.85_As (denoted as α) is presented in Figure 4a. Obviously, the absorption of In_0.15_Ga_0.85_As is almost zero when the photon energy is less than 0.5 eV, which means that the cut-off wavelength is about 2500 nm. This result agrees with the result that electrons with a photon energy lower than the band gap energy cannot be absorbed in the emission layer. Since the In_0.15_Ga_0.85_As photocathode is designed for the extended infrared region, the absorption coefficient of In_0.15_Ga_0.85_As in the NIR region (with a photon energy of 0.5–1.58 eV) can be obtained at approximately ~10^4^ cm^−1^, which is several times higher than GaAs and InAs [35]. Additionally, the absorption coefficient of the Al_0.63_Ga_0.37_As buffer layer (denoted as β) is shown in Figure 4b, which illustrates the absorption peak is blue shifted and also shows an increasing trend with the growing photon energy. The reflectivity spectra of In_0.15_Ga_0.85_As and Al_0.63_Ga_0.37_As are portrayed in Figure 4c,d, which show metal reflection characteristics near the NIR region. The average reflectivity of In_0.15_Ga_0.85_As is lower than that of Al_0.63_Ga_0.37_As. 

### 2.5. Time Response Characteristics of the In_0.15_Ga_0.85_As Photocathode

According to Spicer’s three-step model [29], photoelectrons are excited to the conduction band, then transported towards the band bending region and finally tunnelled through the surface barrier. By solving the photoelectron continuity equation and boundary conditions of the electron transport process, it was deduced that the response time of the In_0.15_Ga_0.85_As photocathode is mainly affected by the electron diffusion coefficient (D_n_), the back interface recombination velocity (S_v_), and the thickness of the emission layer (T_e_), as described in the method. It is difficult to make accurate estimations of the experimental control in the In_0.15_Ga_0.85_As photocathode. Herein, the surface electron escape probability (P) of the photocathode of the emission layer is set to be 0.32 by cubic interpolation [38]. The absorption coefficient of the emission layer can be derived from Figure 4a. The reflectivity of the photocathode (R) is calculated by using optical film matrix theory, which reports a consistent trend in InGaAs-based devices, and hence the reflectivity value is fixed at 0.3 as well [27]. In order to study the time response of the In_0.15_Ga_0.85_As photocathode in the NIR region, the incident light is selected as a Gauss pulse with a wavelength of 1064 nm, and all photoelectron densities emitted by the In_0.15_Ga_0.85_As photocathode were normalized to the incident signal. Under the premise of keeping other parameters unchanged, the influence of the intrinsic time response characteristics with a specific element can be investigated. All the parameters utilized in the simulations are listed in Table 1.

**Figure 5:**
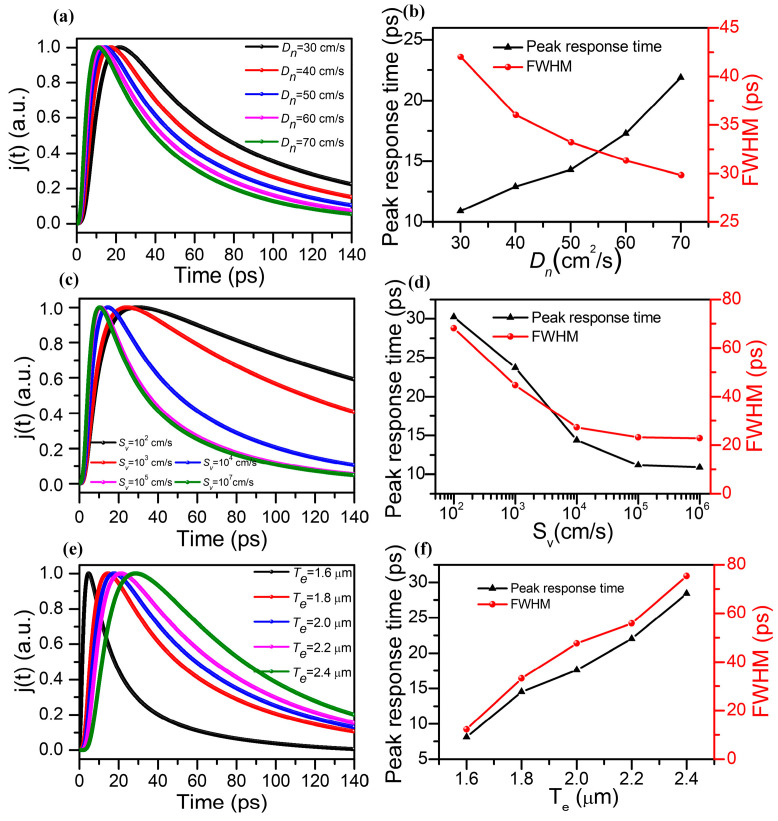
(**a**) The normalized time response and (**b**) the full width at half maximum (FWHM) of the In_0.15_Ga_0.63_As photocathode at different photoelectron diffusion coefficients. (**c**) The normalized time response and (**d**) FWHM at different back interface recombination velocities. (**e**) The normalized time response and (**f**) FWHM at various thicknesses of the emission layer.

**Figure 6:**
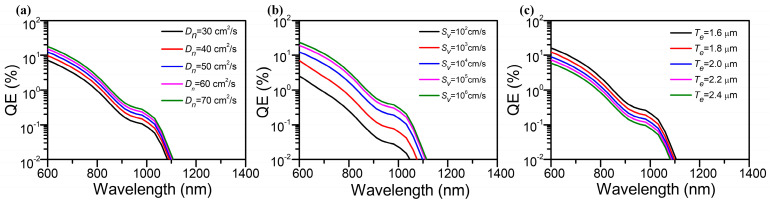
Simulated quantum efficiency curves for the transmission mode In_0.15_Ga_0.63_As-based photocathode by changing (**a**) the electron diffusion coefficient, (**b**) the back interface recombination velocity, and (**c**) the thickness of the emission layer.

Figure 5a depicts the dependency of the photoelectron density emitted by the In_0.15_Ga_0.85_As photocathode on response time with different D_n_ values. In order to highlight the response time of the In_0.15_Ga_0.85_As photocathode with different D_n_ values, the thickness of the emission layer was fixed at 1.8 μm and the back interface recombination velocity was set to 10^4^ cm/s. As shown in Figure 6a, the time duration for the photoelectron density to reach the maximum becomes faster when the electron diffusion coefficient of the In_0.15_Ga_0.85_As photocathode increases from 30 cm^2^/s to 70 cm^2^/s. Meanwhile, owing to the photoelectron diffusion coefficient representing the velocity of photoelectron diffusion moving from high concentration to low concentration driven by the concentration gradient, D_n_ can be equivalently viewed as the photoelectron density under the unit gradient of concentration. That is to say, the higher the diffusion coefficient of the photoelectrons, the faster the diffusion rate of photoelectrons, which increases the energy loss caused by the phonon collision process. Therefore, the decay rate of the photocathode response time increases with higher photoelectron diffusion coefficients. Figure 5b shows the full width at half maximum (FWHM) of the In_0.15_Ga_0.85_As photocathode with different D_n_ values. It is evident that the response time of the In_0.15_Ga_0.85_As photocathode is 29.1 ps when the photoelectron diffusion length is 70 cm^2^/s.

It is known that the surface states (i.e., surface energy levels) of semiconductor materials, similar to the deeper energy levels within the material, can be used as recombination centers to exert the recombination effect on carriers [39]. Therefore, the interfacial recombination process of carriers in semiconductors through surface states can be implemented by defects which are considered as centers of the recombination process. The strength of the interface recombination is characterized by the interface recombination velocity, which is equivalent to the carriers flowing out of the semiconductor surface at a certain speed. Assuming that the thickness of the emission layer is 1.8 μm and the photoelectron diffusion coefficient is 50 cm^2^/s, the normalized time response curves of the In_0.15_Ga_0.85_As photocathode with different back interface recombination velocities are given in Figure 5c. It can be observed that with S_v_ increasing from 10^2^ cm/s to 10^6^ cm/s, the response time to obtain the peak photoelectron density of the In_0.15_Ga_0.85_As photocathode shortens significantly, and the decay rate of photoelectron density also enhances with increasing S_v_. These results can be attributed to the enhancement in the recombination rate of the back interface, which causes more photoelectrons to move towards the recombination center and further alters the concentration distribution of photoelectrons. This phenomenon will not only help to improve the free diffusion motion of photoelectrons but also change the band structure of the interface between the emission layer and buffer layer, which results in a reduction in the potential barrier at the interface. The FWHM of the In_0.15_Ga_0.85_As photocathode with different S_v_ values is given in Figure 5d, which demonstrates that the time response of the In_0.15_Ga_0.85_As photocathode is 14.9 ps when the interface recombination rate further increases to 10^5^ cm/s and almost reaches saturation.

It is obvious that the thickness of the emission layer also determines the photoemission process of the photocathode and the energy loss caused by electron collisions. Supposing that the photoelectron diffusion coefficient is 50 cm^2^/s and the back interface recombination velocity is fixed is 10^4^ cm/s, Figure 5e,f shows the normalized time response and FWHM at various thicknesses of the emission layer. The response time of the In_0.15_Ga_0.85_As photocathode increases from 6.1 ps to 28.4 ps when T_e_ grows from 1.6 μm to 2.4 μm because the transport distance of photoelectrons increases, further inducing photoelectron energy loss. It is worth noting that the effect of thickness on the In_0.15_Ga_0.85_As photocathode shows a similar trend to the GaAs-based photocathode [40]. However, due to the large absorption coefficient of In_0.15_Ga_0.85_As in the NIR region, more photoelectrons can be excited to the conduction band, which is beneficial for the photoemission process of the In_0.15_Ga_0.85_As photocathode. Meanwhile, the FWHM of the In_0.15_Ga_0.85_As photocathode is 10.2 ps at a thickness of 1.6 μm, which is much lower than that of the traditional GaAs photocathode (~69 ps at a wavelength of 860 nm) [14] and InP/InGaAs (~35 ps at 1550 nm) [41], and can meet the ultrafast response requirements of imaging intensifiers and atomic lifetime detectors in the NIR region.

### 2.6. Quantum Efficiency of the In_0.15_Ga_0.63_As Photocathode

In addition, the quantum efficiency can also be calculated and altered by the electron diffusion coefficient, the back interface recombination velocity, and the thickness of the emission layer. Hence, we also analyzed the impact factors of the quantum efficiency, including D_n_, S_v_, and T_e_. Related to the properties of the material, the thickness of the buffer layer was set as 0.2 μm [23] and the electron diffusion length was 1.7 μm [27]. By fixing S_v_ at 10^4^ cm/s and T_e_ at 1.8 um, the quantum efficiency curves of the proposed structure with different electron diffusion coefficients of In_0.15_Ga_0.85_As are simulated for optimal performance of the photocathode, as given in Figure 6a. The results indicate a higher QE of the In_0.15_Ga_0.63_As photocathode in the NIR region compared with the previous InGaAs-based photocathode (~0.01% at 1064 nm) [38], and it redshifts with the increasing D_n_. It is noted that the QE at wavelengths less than 1100 nm rises, which is ascribed to the high motion speed of photoelectrons in the emission layer. According to the absorption intensity distribution in Figure 4, due to the In_0.15_Ga_0.85_As emission layer and the Al_0.63_Ga_0.37_As buffer layer having no ability to absorb light with a photon energy less than 1.13 eV (corresponding to the cut-off wavelength at around 1100 nm), the quantum efficiency is approximately zero. 

Similarly, the simulated quantum efficiency curves with varying back interface recombination velocities are given in Figure 6b, while the D_n_ and T_e_ remain unchanged. It is obvious that the quantum efficiency increases with the growing back interface recombination velocity. With the enhancement in S_v_, plenty of photoelectrons recombine at the back interface and are transported to the emission layer rather than back into the buffer layer due to the existence of a potential barrier at the back interface, which accounts for promoting the QE. Furthermore, supposing that S_v_ and D_n_ are 10^4^ cm^2^/s and 50 cm/s, Figure 6c exhibits the changes in the theoretical quantum efficiency with different thicknesses of the emission layer. It turns out that the QE shows a declining trend with the growing thickness of the emission layer. For incident light with a photon energy that can be fully absorbed in the emission layer, the T_e_ of the In_0.15_Ga_0.85_As emission layer increases the transport distance of the excited electrons, which decreases the quantum efficiency. 

## 3. Methods

### 3.1. First Principles Calculations of In_0.15_Ga_0.85_As and Al_0.63_Ga_0.37_As

First principles calculations, based on density functional theory (DFT), were carried out to optimize the structures and calculate the related electronic properties using the Vienna Ab initio Simulation Package (VASP) [42]. The projector augmented wave (PAW) method and the Perdew–Burke–Ernzerhof (PBE) functional of the generalized gradient approximation were adopted for the electron–ion interaction and exchange–correlation functional, respectively [43,44]. The valence electron configurations in reciprocal space are 4d^10^5s^2^5p^1^, 3d^10^4s^2^4p^1^, 3s^2^3p^1^, and 4s^2^4p^3^ corresponding to In, Ga, Al, and As elements, respectively. The cutoff energy was set as 420 eV. All models were optimized until the convergence accuracy was below 10^−6^ eV/atom and the interatomic force was less than 0.01 eV/Å. The maximum atomic displacement during the iterations was less than 10^−4^ nm. 

### 3.2. Calculation Methods for Photoemission Performance of the In_0.15_Ga_0.85_As Photocathode

**Figure 7:**
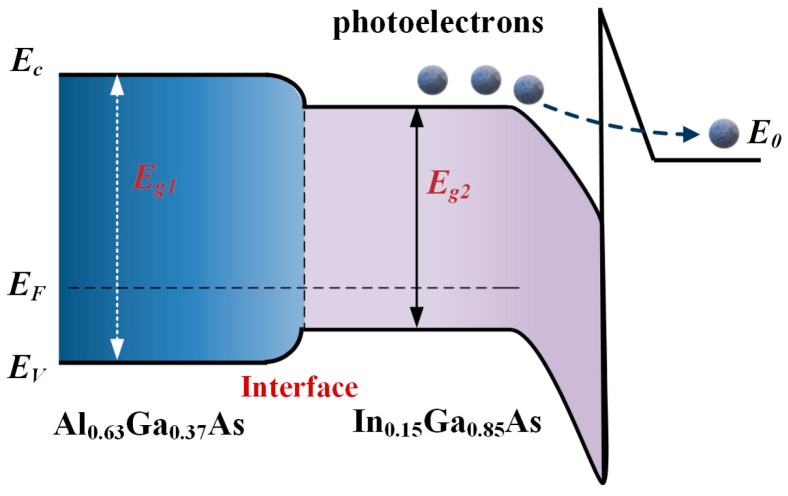
A schematic of the energy band structure of the In_0.15_Ga_0.85_As photocathode.

According to Spicer’s three-step model [45], the photoemission process of the In_0.15_Ga_0.85_As photocathode can be well described. In order to intuitively describe the electron transport process, the energy band structure diagram of the proposed In_0.15_Ga_0.85_As photocathode is portrayed in Figure 7. When the incident photon energy is larger than the band gap of In_0.15_Ga_0.85_As, it can be absorbed by the emission layer and electrons in the valence band are transported to the conduction band. This process is affected by the absorption coefficient of the emission layer. Subsequently, the photoelectrons from the bulk move towards both the surface of the emission layer and the interface by the diffusion process. Only photoelectrons near the surface of the emission layer are able to tunnel through the potential barrier and finally escape into the vacuum. It is worth noting that when the emission layer is thin enough, the high-energy photons can also be absorbed by the buffer layer as well. Meanwhile, due to the drift of the electric field, some photoelectrons excited near the back surface of the Al_0.63_Ga_0.37_As buffer layer are recombined at the interface, and others will be transported into the emission layer rather than back into the buffer layer due to the existence of a potential barrier at the back interface. Therefore, the diffusion process of photoelectrons will obviously exhibit the relaxation phenomenon.

According to the photoemission process of the In_0.15_Ga_0.85_As photocathode, the photoelectrons escape from the emission layer mainly by diffusion, which can be considered as a dynamic function of time (t). The basic electron transport equation is given by [46]:

(1) $$\frac{\partial \Delta n(x,t)}{\partial t}=D_{n}\frac{\partial^{2}\Delta n(x,t)}{\partial x^{2}}-\frac{\Delta n(x,t)}{\tau_{}}\;x\in [0,T_{e}]$$

where Δn represents the concentration of photoelectrons, D_n_ is the diffusion coefficient of photoelectrons in the emission layer, T_e_ is the thickness of the emission layer, x is the distance from the interface between the emission layer and buffer layer, and τ is the lifetime of photoelectrons in the emission layer.

When defining the boundary conditions of Equation (1), the electron contribution from the buffer layer should be considered, and we assume that the photocathode is illuminated under a light source which can be expressed by I_0_ δ(t) at t = 0. Accordingly, the one-dimensional continuity equation and boundary conditions which are suitable for the In_x_Ga_1−x_As-based photocathode are given by:

(2) D∂Δn∂xn−SνΔnx=0=0

(3) $$\Delta n(T_{e},t)=0$$

(4) $$\Delta n(x,0)=P(1-R)\cdot \alpha \cdot e^{-\alpha x}\cdot I_{0}\cdot \delta (t)$$

where S_v_ is the back interface recombination velocity of the interface, α is the absorption coefficient of the emission layer, P is the electron escape probability, and R is the reflectivity of the surface of the photocathode.

According to Equations (1)–(4), the numerical solution of the photocurrent density in the NEA photocathode can be obtained by matrix difference method:

(5) $${\left.j(t)=-PD_{n}\cdot \frac{\partial \Delta n(x,t)}{\partial x}\right|}_{x=T_{e}}$$

In addition, by taking into account multilayer optical thin film theory [47] and the one-dimensional continuity equations which stand for the photoelectrons in the buffer layer [48], the formula for the quantum efficiency Y (defined as: Y = j/I_0_) of the transmission mode In_0.15_Ga_0.85_As photocathode can be finally solved as follows:

(6) $$\begin{array}{l} Y(h\nu )=\frac{P(1-R_{})\alpha_{h\nu }L_{d}\exp(-\beta_{h\nu }T_{b})}{\alpha_{h\nu }{}^{2}L_{d}{}^{2}-1}\times \{\frac{\alpha D_{n}+S_{v}}{\left(D_{n}/L_{d})\cosh(T_{e}/L_{d})+S_{v}\sinh(T_{e}/L_{d}\right)}-\\ \frac{\exp(-\alpha_{h\nu }T_{e})[S_{v}\cosh(T_{e}/L_{d})+(D_{n}/L_{d})\sinh(T_{e}/L_{d})]}{\left(D_{n}/L_{d})\cosh(T_{e}/L_{d})+S_{v}\sinh(T_{e}/L_{d}\right)}-\alpha L_{d}\exp(-\alpha_{h\nu }T_{e})\} \end{array}$$

where L_d_ is the electron diffusion length, β is the absorption coefficient of the buffer layer, and T_b_ is the thickness of the buffer layer.

## 4. Conclusions

In this paper, the energy band, electronic structure, and optical properties of In_0.15_Ga_0.85_As are calculated based on DFT. The time response characteristics of photoelectron dynamics in the In_0.15_Ga_0.85_As photocathode emission layer in the near-infrared region are investigated by varying the parameters of the emission layer. An enhanced time response of the In_0.15_Ga_0.85_As photocathode can be obtained with increases in the photoelectron diffusion coefficient and the back interface recombination velocity. By reducing the thickness of the emission layer, the response time of the In_0.15_Ga_0.85_As photocathode can reach a minimum value of 6.1 ps, which is much lower than that of the GaAs photocathode. The high quantum efficiency of the transmission mode In_0.15_Ga_0.85_As photocathode can be obtained by optimizing the parameters of the emission layer as well. Our work opens new doors for designing a high-performance NEA photocathode in the NIR region and helps us to understand the photoemission mechanism of other photocathodes.

## Figures and Tables

**Table 1:** The simulated parameters of photoemission properties for the transmission mode In_0.15_Ga_0.85_As photocathode.

Parameter	Value	Description	References
D_n_ (cm^2^/s)	30–70	Electron diffusion coefficient	This work
S_v_ (m/s)	10^2^–10^6^	Back interface recombination velocity	This work
T_e_ (μm)	1.6–2.4	Thickness of the emission layer	This work
α (cm^−1^)	2.16 × 10^4^ (at 1064 nm)	Absorption coefficient of the emission layer	This work
β (cm^−1^)	1.35 × 10^4^ (at 1064 nm)	Absorption coefficient of the buffer layer	This work
P	0.32	Surface electron escape probability	[38]
R	0.3	Reflectivity of the photocathode	[30]
L_d_ (μm)	1.7	Diffusion length	[30]
T_b_ (μm)	0.2	Thickness of the buffer layer	[25]

## Data Availability

Not applicable.
